# Filtered pose graph for efficient kinect pose reconstruction

**DOI:** 10.1007/s11042-016-3546-4

**Published:** 2016-05-13

**Authors:** Pierre Plantard, Hubert P. H. Shum, Franck Multon

**Affiliations:** 1 FAURECIA Automotive Seating, Etampes, France; 2 University Rennes 2, Rennes, France; 3 Northumbria University, Newcastle, UK; 4 Inria, Rennes, France

**Keywords:** Kinect, Pose reconstruction, Occlusion, Motion analysis

## Abstract

Being marker-free and calibration free, Microsoft Kinect is nowadays widely used in many motion-based applications, such as user training for complex industrial tasks and ergonomics pose evaluation. The major problem of Kinect is the placement requirement to obtain accurate poses, as well as its weakness against occlusions. To improve the robustness of Kinect in interactive motion-based applications, real-time data-driven pose reconstruction has been proposed. The idea is to utilize a database of accurately captured human poses as a prior to optimize the Kinect recognized ones, in order to estimate the true poses performed by the user. The key research problem is to identify the most relevant poses in the database for accurate and efficient reconstruction. In this paper, we propose a new pose reconstruction method based on modelling the pose database with a structure called Filtered Pose Graph, which indicates the intrinsic correspondence between poses. Such a graph not only speeds up the database poses selection process, but also improves the relevance of the selected poses for higher quality reconstruction. We apply the proposed method in a challenging environment of industrial context that involves sub-optimal Kinect placement and a large amount of occlusion. Experimental results show that our real-time system reconstructs Kinect poses more accurately than existing methods.

## Introduction

Immersive environments with natural movement interaction have become popular in human performance training and analysis, as they provide standardized training environments and objective evaluations [5]. In these applications, it is important to capture the user’s motion accurately and efficiently with minimal intrusion and technological constraints. Microsoft Kinect can measure 3D human motion without complex setup and wearable devices, which makes it a promising system for motion analysis in such a context. Nowadays, low-cost Kinect has been applied to clinical gait analysis [2, 3, 16], sign-language analysis [17, 24], sport training [7, 22] and ergonomics [12, 39].

However, using Kinect in complex environments, with sub-optimal camera placements and cluttered environments, has not been tested. Kinect is designed to be used in open areas and should be placed directly in front of the user. Accuracy of the Kinect skeleton data drops when these conditions are not satisfied. However such constraints cannot be easily satisfied in real sports or industrial context with machines, equipment and many other objects or people that clutter the environment. Moreover, since Kinect recognizes body parts based on the observed features in the depth image [35], partial or total occlusion strongly affects the recognition rate [30]. Especially, using Kinect in an industrial environment where users have to handle large equipment is therefore challenging due to the large amount of occlusion.

To improve the robustness of Kinect under complex environment, an effective method is to correct potential errors by reconstructing the unreliable part of the Kinect poses using prior knowledge of human movement [37]. The idea is to construct a database of accurately captured human poses as a prior to optimize the Kinect ones, so as to estimate the true pose performed by the user despite the errors returned by the Kinect. Here, the key research problem is to identify the most relevant subset of poses in the database to accurately and efficiently reconstruct skeleton data. In particular, the brute force database search proposed by Shum et al. [37] cannot ensure temporal continuity across frames. Thus, it reduces the relevance of the retrieved poses and therefore degrades the quality of the reconstruction.

In this paper, we introduce a data structure named Filtered Pose Graph to enhance the pose reconstruction process introduced by [37]. Using Filtered Pose Graph aims at ensuring continuity and relevance in the candidate selection, and consequently enhance the performance of the reconstruction. Hence, with such a graph, a more relevant set of poses can be selected with smaller computational time.

We have two main contributions in the paper:

- As an offline process, we propose a Filtered Pose Graph structure to organize a database of poses in order to enhance the performance of the online pose reconstruction process. Nodes are representative poses in the database and edges are potential transitions between two poses ensuring continuity if connected one to each other.
- As an online process, we propose a customized search algorithm based on the Filtered Pose Graph to efficiently retrieve the set of poses that are relevant depending on the current pose before reconstruction. This not only ensures continuity in the retrieved poses, but also greatly enhances computation cost to reach real-time perform during reconstruction for interactive applications.

To challenge the system, we carried-out a set of experiments under challenging situations similar to real industrial environments: occlusions and sub-optimal camera placement. Results show that our method generated higher quality reconstructed poses than previous works, such as the original Kinect pose estimation [35] and the reconstruction method proposed in [37].

The paper is organized as follows. Section 2 presents a review of previous works. Then, Section 3 describes the overview of our approach. Section 4 explains how the database is structured as a graph to speed up the process and improve the quality of the reconstruction. Sections 5, 6 and 7 provide information about reliability estimation, example-based optimization and dynamic filtering of the results respectively. Experiments performed under highly constrained industrial scenarios are presented in Section 8. Finally, we conclude and discuss our method in Section 9.

## Related works

Firstly, in this section, we review previous works on motion analysis based on depth cameras. Then, we focus on the pose reconstruction based on incomplete and noisy data. We finally review and discuss the data structures used in computer animation to organize databases of motion clips.

### Depth camera based motion analysis

Motion sensing systems based on depth camera, such as the Kinect, use pattern recognition techniques based on depth images to recognize and estimate a human pose. In particular, Kinect recognizes different body parts using decision forests trained with a large number of synthesized depth images [35]. Depth images can be viewed as 2.5D point clouds, and fusing them can generate a 3D mesh of the tracked object [29]. Real-time performance capture involving both movement and surface deformation has become possible thanks to this approach [43]. Depth cameras have introduced a new direction of motion analysis research and a new generation of motion-based application that has not been seen before [20].

There is a large body of research aiming at estimating the accuracy of the Kinect data for various types of applications, such as motion analysis, rehabilitation and ergonomics. Previous works reported good accuracy of the Kinect when analysing simple full body motion such as gait [11], or reaching movements [10]. It has also shown high accuracy in terms of timing and range during large movements, with potential application in clinical assessment of Parkinson’s diseases [16] and in rehabilitation [14]. Automatic pose evaluation system with Kinect has shown consistent results in ergonomics for musculoskeletal disorder risk assessment, such as the Rapid Upper Limb Assessment (RULA) [30], which is a popular assessment method in ergonomics [28]. Kinect is particularly suitable for pose monitoring in workplace to avoid musculoskeletal injury due to its small size and easy setup [13]. It can be used for real-time ergonomic analysis of lifts performed by human so as to minimize injury [26]. Combing colour and depth information can enhance the tracking in smart environments [19]. However all these methods have been tested in laboratory condition, whereas actual work condition exhibit challenging constraints, such as sensor placement and cluttered environments.

### Pose reconstruction

The major problem for real-time motion analysis is the completeness and accuracy of the input data. Raw Kinect data exhibits high inaccuracies mainly due to sub-optimal sensor placement and occlusions [30]. Using the knowledge provided by a motion database, it is possible to reconstruct high dimensional full-body movements based on low dimensional input. Hence, considering the local subspace in the database where the poses match well with the joint positions from a small number of reflective markers, it is possible to estimate the position of all the other body joints [8]. Since human motion is highly non-linear, learning statistical dynamic models as a motion prior can produce movements that better satisfy the required constraints [9]. Such a motion prior concept is applied to real-time pose reconstruction by generating higher quality movements [25]. When adapting this idea to accelerometer-based systems, an online lazy neighbourhood graph is used to minimize false positive samples in the local subspace [38]. When applying these methods to reconstruct Kinect poses, the main problem is that they assume the input data to be accurate, whereas the joint positions estimated by Kinect are noisy or even incorrect due to sensor error and occlusion.

While basic inaccuracy such as jittery movements can be corrected by physical filters [36], more complex problems such as incorrectly detected joints cannot be corrected with this approach. Methods based on optimizing a set of spatiotemporal constraints have been proposed to address this type of error [33]. To this end, information about the reliability of the current Kinect pose is necessary. Such a reliability estimation can then be integrated into a lazy learning framework to reconstruct the true pose [37]. Since the learning framework requires a large amount of poses, Gaussian Process is proposed to produce an abstract representation of the pose space and reduce the required database size [42]. However the unstructured nature of the database used during optimization cannot guarantee continuity of the resulting reconstructed poses and leads to consider a large number of candidate, some of them being inappropriate. In this paper, we propose a new data structure, named Filtered Pose Graph, to preselect the most relevant subset of poses to consequently enhance the global performance of the reconstruction method.

### Motion database structure

With an appropriate motion database structure, we assumed to enhance optimization-based reconstruction systems, as described in the previous section. Motion graphs have been introduced in computer animation to organize a database of motion as a set of nodes (indicating poses or small clips) and edges (indicating possible connection between nodes without discontinuity) [23]. It has been used extensively to automatically combine motion clips to produce and control character animations [18, 31, 32]. A large motion database usually results in a dense graph with a large number of nodes and edges. As a result, several authors proposed to decrease the size of the motion graph [41] or synthesize new artificial motion and consequently to improve graph connectivity [40]. Parametric motion graphs have been introduced to support motion re-sequencing and motion blending for parameterized movements [21]. Fat graphs combine similar poses into fat nodes and modelling correspondence between fat nodes as fat edges [34]. Motion-motif search [4] could be used to enhance the performance and the quality of the search of similar motions in large databases.

All these previous structures have been designed to identify and represent similar poses in a database to control character animation. The main point in character animation using this approach is to find a sequence of poses/clips that minimizes a distance to a goal, such as following an imposed trajectory while preserving continuity. In a pose reconstruction perspective, the problem is different: the data structure should enable us to select examples that would help to reconstruct poses with reliable and unreliable joints. Hence, variability is important to guarantee that a variety of examples is available for a given pose with unreliable parts, whereas computer animation approaches generally tend to gather similar examples in a unique node.

## System overview

**Fig. 1:**
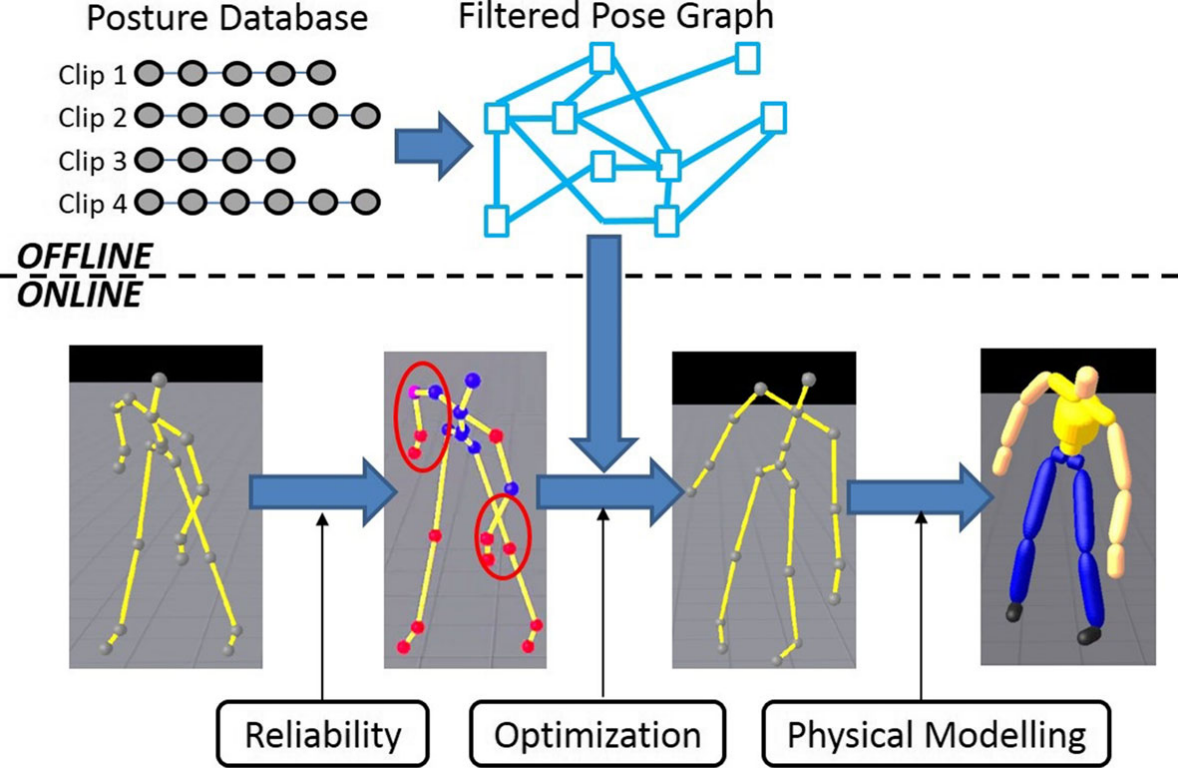
Overview of our Kinect pose reconstruction system

The overview of our system is shown in Fig. 1. Offline, a human pose database is constructed using accurately captured human motion obtained thanks to an optical motion capture system. Then, the database is organized as a Filtered Pose Graph aiming at enhancing the performance of the reconstruction. Online, reliability of the current Kinect pose is evaluated for each individual joint. We then use the Filtered Pose Graph to preselect candidate poses prior to an optimization process aiming at replacing unreliable joint position by more appropriate ones. Finally, a physical model is used to filter the results, ensuring continuity and physical correctness of the resulting motion.

## Database organization with filtered pose graph

In this section, we explain how we capture accurate motion using an optical motion capture in an industrial operation context, and detail the method to create the Filtered Pose Graph. Notice that while the content of our database is in industrial context, our methodology can be applied for any type of motion, but industrial context provides us with very challenging constraints due to cluttered environments.

### Motion data collection

In this section, we describe the protocol and methods used to capture motion which will serve as examples in the Filtered Pose Graph. As these examples need to be error-free, we used an accurate optical motion capture system (Vicon, product of Oxford Metrics). To be useful for Kinect data improvement, such motions should 1) belong to the same family of tasks as those performed by the user during run-time, and 2) contain enough relevant examples to provide reasonable variability. The former constraint ensures that the system has enough information to reconstruct the run-time pose, while the latter ensures to get examples with different styles for the same task.

Since our main target application is to monitor industrial operations, we captured the motion of trained operators when performing a series of short working tasks. The list of motion is designed according to Method Time Measurement (MTM) list [27], which is commonly used in industrial settings to analyse tasks performed by operators. Using the Vicon system, we captured 130 types of motion including grasping, re-grasping, putting, moving, position, etc. as suggested in MTM. For each type of motion, the operator performed 5 trials, with different speeds and locations to ensure variability. The captured motion of the different operators were then retargeted to the Kinect skeleton structure using commercial software. We normalized individual pose by removing the rotation along the vertical axis and the global 3D translation, as such information is irrelevant to the pose context. Each pose is represented as a set of joint positions *p*={*j*(*x*_*j*_,*y*_*j*_,*z*_*j*_)}_*j*=1..*N*_, where *N* is the number of joints in the pose, and *x*_*j*_,*y*_*j*_,*z*_*j*_ are the 3D Cartesian coordinates of the *j* ^*th*^ joint.

### Graph construction

The Filtered Pose Graph is computed using an unorganized set of recorded motions. We build the Filtered Pose Graph using a two-steps process as described below.

In the first step, to reduce the size of the graph and consequently save computation time, we perform an intra-motion and inter-motion filter to remove redundancy or too-similar poses. Two poses are supposed to be similar if the distance in-between is lower than a given threshold. This distance *d* *i* *s* *t*(*p*_*a*_,*p*_*b*_) between two poses *p*_*a*_ and *p*_*b*_ is defined as the maximum of all the joint position differences between *p*_*a*_ and *p*_*b*_:

1 $$ dist(p_{a}, p_{b})=max_{j=1..N}\parallel p_{a}(j)-p_{b}(j)\parallel  $$

where *N* is the total number of joints. Contrary to many traditional approaches based on the mean error between the joints, using the maximum error enables us to detect large local differences in one particular joint. In particular, since our motion database is specific in industrial operations, many motions are different in terms of the arm movement only. Traditional methods of averaging the errors of all joints may be inefficient to detect such differences in some cases.

**Fig. 2:**
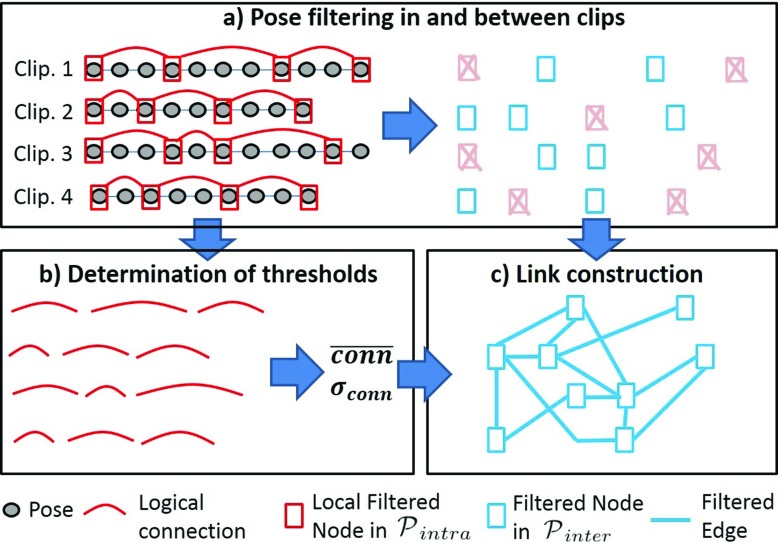
Filtered Pose Graph construction. (*Upper Left*) Pruning similar poses within each clip to create local Filtered Nodes in $\mathcal {P}_{intra}$ (*red nodes*). (*Upper Right*) Pruning similar Local Filtered Nodes in $\mathcal {P}_{intra}$ to obtain $\mathcal {P}_{inter}$ (*blue Filtered Nodes*). (*Lower Left*) Determining statistical information about the natural links between successors in $\mathcal {P}_{intra}$ within each clip. (*Lower Right*) Combining logical connections between nodes in $\mathcal {P}_{inter}$ as Filtered Edges


The intra-motion filter is performed by screening the motion from start to end, and keeping only the poses that are not similar to the previous ones (i.e. *dist* was less than a threshold *t* *h* *r* *e* *s*_1_), as shown in Algorithm 1. The upper left part of Fig. 2 depicts this filtering process starting from input poses (grey circles) to a set of Local Filtered Nodes named $\mathcal {P}_{intra}$ (red boxes) within each clip. Then, inter-motion filter is performed using the same process to eliminate similar Local Filtered Nodes between motions, as shown in Algorithm 2. The red boxes with a cross in the upper right part of Fig. 2 represent local Filtered Nodes that have been eliminated because too similar to previous ones. The resulting set of Filtered Nodes (blue boxes) is denoted $\mathcal {P}_{inter}$. After both local and global filtering, we obtain a compact set of nodes in which poses are at least separated by distance *t* *h* *r* *e* *s*_1_ on at least one of the joints.

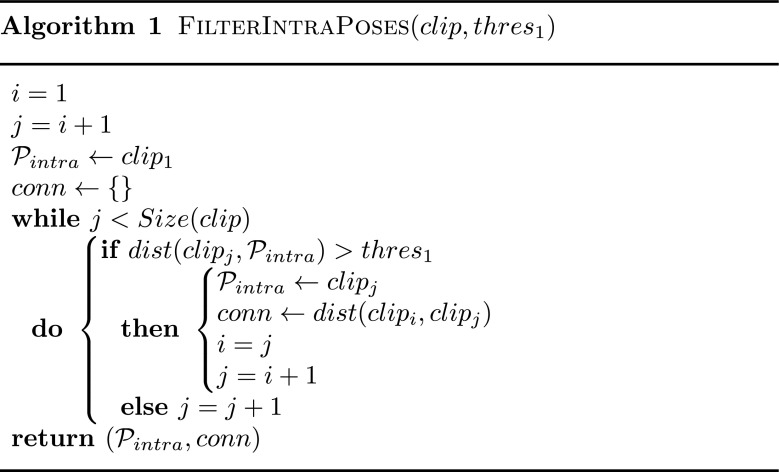
 
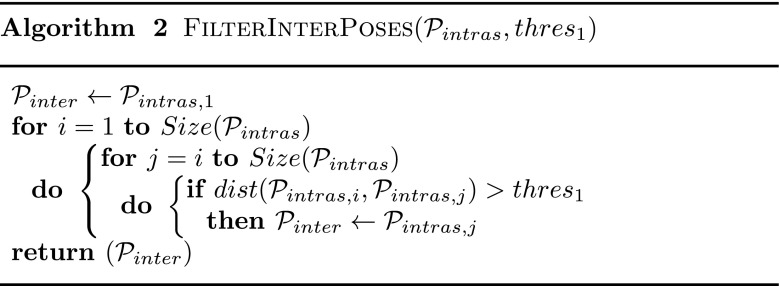


In the second step, we compute the Filtered Edges to connect Filtered Nodes and build the final Filtered Pose Graph. In a motion, two successive Local Filtered Nodes $\mathcal {P}_{intra}$ are naturally connected as they correspond to an existing sequence of poses. Firstly, we consequently identify these logical connections within a clip, which are illustrated with the red curves in the lower left part of the Fig. 2. For each connection, Equation 1 is used to compute the distance between the two connected nodes $\mathcal {P}_{intra}$. The average distance $\overline {conn}$, and the standard deviation *σ*_*conn*_ are computed for all these existing connections within each clip. Such values are then used to estimate a reasonable distance threshold *t* *h* *r* *e* *s*_2_ below which two nodes can be logically connected within or in-between clips without discontinuity:

2 $$ thres_{2}=\overline{conn} + 2\sigma_{conn} $$

Finally, we compute all the distances between all the Filter Nodes $\mathcal {P}_{inter}$ within and between clips and created a Filtered Edge if this distance is smaller than *t* *h* *r* *e* *s*_2_. The resultant Filtered Edges are depicted with blue lines in the lower right part of Fig. 2. Algorithm 3 shows how Filtered Edges are computed. In our system, Filtered Edges are considered to be bidirectional as we assume motion can be performed in both forward and backward directions. 
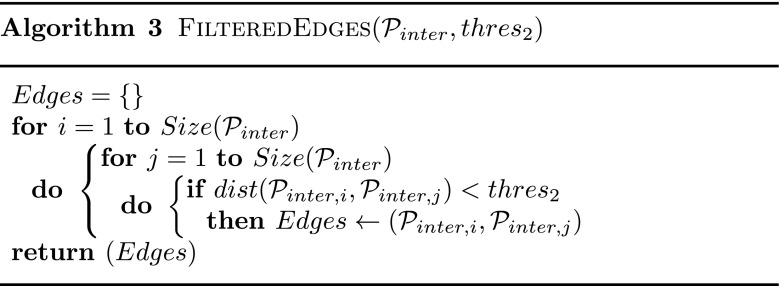


The two-steps process to construct a Filtered Pose Graph is summarized in Algorithm 4. 
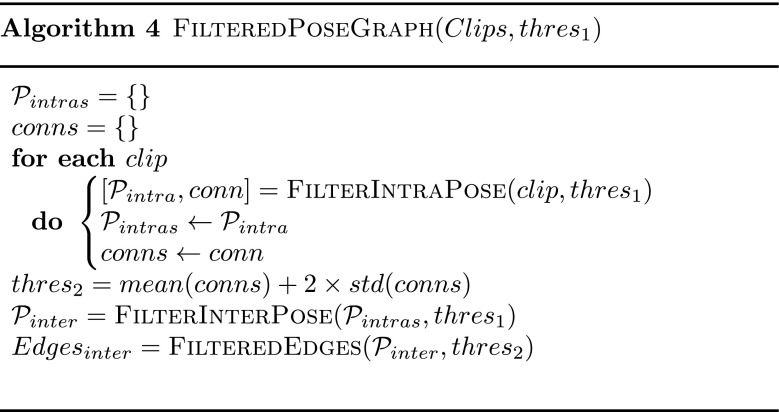


When selecting pose candidates to reconstruct a pose using optimization (see Section 6), it is important to select poses that have a chance to help reconstructing unreliable parts of the body. It helps us to estimate the actual pose of the user while taking into account what the user would logically perform at this time, in a continuous manner.

In our experiment, 532,624 poses were captured. We applied the filtering process to obtain a reduced number of Filtered Nodes equal to 2,048, using *t* *h* *r* *e* *s*_1_=0.2*m*. When applying the above method, we found that $\overline {conn}= 0.21m$ and *σ*_*conn*_=0.02*m* for this database, leading to set *t* *h* *r* *e* *s*_2_ to 0.21+2∗0.02=0.25*m*.

## Joint reliability estimation

The Filtered Pose Graph aims at providing relevant examples to reconstruct unreliable parts of the skeleton returned by the Kinect. Thus, it is important to identify which part of the skeleton is reliable and which part was badly estimated by the Kinect. Indeed, the pose provided by the Kinect may consist of incorrect joints due to occlusions and sensor errors. In this paper, we adapted the method proposed in [37] to evaluate the reliability of each joint in the Kinect skeleton. Reliability is represented as a real number between 0 (not reliable at all) and 1 (fully reliable), and is calculated based on three terms. Here, we briefly review the algorithm.

The first term is the behaviour term that calculates the amount of vibration of a joint, modelled as the acute angle between the displacement vectors formed by joint *j* in the past *f*_*b*_ frames:

3 $$\begin{array}{@{}rcl@{}} Rb(j) = 1-mean_{f=1..f_{b}} \left[ \frac{d_{f-1}(j) \cdot d_{f}(j)}{|d_{f-1}(j)| |d_{f}(j)|} \right] \end{array} $$

where *d*_*f*_(*j*) = *p*_*j*_(*f*)−*p*_*j*_(*f*−1) is the displacement vector of joint *j* from frame *f*−1 to frame *f*. Note that the angle value is set to 0 if the length of either *d*_*f*_(*j*) and *d*_*f*−1_(*j*) is smaller than a threshold, to avoid large angle changes when unnoticeable vibrations occur. *R* *b*(*j*) is truncated between 0 and 1 if it is outside this interval. The behaviour term is designed according to the fact that human movement must be continuous. This term is useful to detect dynamic errors, when a joint vibrates around the actual position due to occlusion.

The second term is the bone length consistency term that evaluates if bone length remains constant during the motion:

4 $$\begin{array}{@{}rcl@{}} Rc(j) = 1-mean_{b=1..b_{total}} \left[\frac{|l_{b}(f)-l_{b\_ref}|}{l_{b\_ref}} \right] \end{array} $$

where *b*_*total*_ is the total number of bones connecting joint *j*, *l*_*b*_(*f*) and $l_{b\_ref}$ are the observed bone length of bone *b* at frame *f* and the reference bone length value respectively. The reference length of all bones is computed based on measured joint positions using a pose where all the joints are supposed to be perfectly visible. *R* *c*(*j*) is truncated between 0 and 1 if it goes beyond this interval.

The third term is the Kinect feedback term, *R* *f*(*j*), that is calculated based on the joint tracking state returned by the Kinect for each joint *j*. It is set to 1.0 if this tracking state is “tracked”, and 0.0 if the state is either “inferred” or “not tracked”.

The reliability of the joint *j* is defined as a combination of these three terms:

5 $$\begin{array}{@{}rcl@{}} R(j) = \min(Rb(j) \text{, } Rc(j) \text{, } Rf(j)) \end{array} $$

A Gaussian filter is then applied to the resulting reliability values using previously computed ones, to ensure continuity.

The reliability terms measure the joint reliability for different types of error. The behaviour term is useful to detect dynamic error, when a joint vibrates around the true position due to occlusion. The bone length term is useful to detect other type of errors that make the distance between two joints of the same segment change over time. The Kinect feedback term provides information delivered directly by the Kinect to state if the joint is tracked or inferred because occluded or outside of the capture area. As a result, depending on the type of error, one or more reliability terms may be sensitive, while the others may not. Using the minimum value instead of the average ensures the system to capture all types of errors.

## Pose reconstruction

The aim of this process is to reconstruct unreliable parts of the body that are considered to be badly estimated by the Kinect. To this end, we use a local optimization process consisting of two main steps: selecting the pose candidates which are considered as relevant according to the current Kinect pose, and optimizing the pose based on a set of energy functions. The main contribution of this paper is to design a novel pose selection algorithm to enhance the performance of the optimization process introduced in previous works [37].

### Database pose evaluation

Selecting potential candidates in the Filtered Pose Graph based on the pose delivered by the Kinect involves defining a dedicated metric. This metric aims at comparing the input Kinect pose and all the potential candidates (Filtered Nodes) to find good candidates before reconstruction. In previous works [37] this distance metric was only based on the similarity (e.g. a distance) between the Kinect pose and each candidate. Similarity could only be tested on reliable joints as the other ones could be totally false. However candidates selected using this criterion only could lead to important discontinuities in the unreliable joints trajectories. We consequently propose to ass another criterion based on continuity to overcome this limitation.

Similar to [37], the first selection evaluation function returns a score related to the similarity between the tested Filtered Node and the current Kinect pose. This score is high when the two poses are similar. Reliability values computed for each joint provided by the Kinect are used as weights, such that reliable joints would have a higher weight when computing this score:

6 $$\begin{array}{@{}rcl@{}} Ss(p_{k}, p_{d}) = mean_{j=1..j_{total}}\left[ R(j) \times (p_{k}(j) - p_{d}(j)) \right] \end{array} $$

where *p*_*k*_(*j*) and *p*_*d*_(*j*) are the observed Kinect position and database position of joint *j* respectively, *R*(*j*) is the reliability value of joint *j* from the Kinect pose.

Only using (6) to select candidates does not take the unreliable parts of the body into account, as explained above. To overcome this problem, another criterion is introduced to ensure continuity for the reliable but also the unreliable joints. To this end, we compute a prediction of the current pose depending on the previous reconstructed one *p*_*l*_ and its derivative $\dot {p}_{l}$:

$$\begin{array}{@{}rcl@{}} Sc(p_{k}, p_{d}) = mean_{j=1..j_{total}} \left[ (1-R(j)) \times (p_{l}(j)+ \dot{p}_{l}(j) \times dt - p_{d}(j)) \right] \end{array} $$

where *p*_*l*_(*j*) and $\dot {p}_{l}(j)$ are the position and velocity of the last reconstructed pose for joint *j* respectively, and *dt* is the frame time. Note that introducing a weight 1−*R*(*j*) enables us to give more importance to non-reliable joints when computing this criterion. The key idea is to consider that reliable joints should be mainly taken into account using the other criterion *S*_*s*_, while continuity is the only available criterion for unreliable joints.

The final score to select a pose *p*_*d*_ is defined as the sum of the two terms:

7 $$\begin{array}{@{}rcl@{}} S(p_{k}, p_{d}) = \left\{ \begin{array}{l} Ss(p_{k}, p_{d})+Sc(p_{k}, p_{d}) \phantom{aaa} \text{if}\, f>1 \\ Ss(p_{k}, p_{d}) \phantom{aaaaaaaaaaaaa}\, \text{otherwise} \end{array} \right. \end{array} $$

where *f* is the frame number, and in frame 1 $\dot {p}_{l}(j)$ is not available.

Let us consider now how these criterions are used to efficiently select appropriate candidates in the Filtered Pose Graph before reconstruction.

### Database pose selection

Before reconstruction, a set of Nodes are selected from the Filtered Pose Graph using (7). Previous works [37] performed a brute force search with an unorganized database, which leads to high computation time and selection of irrelevant Nodes. To overcome this limitation, we take advantage of the Filtered Pose Graph to evaluate Nodes that have high potential to contribute to the pose reconstruction process, thereby enhancing system performance and reducing computation time.

**Fig. 3:**
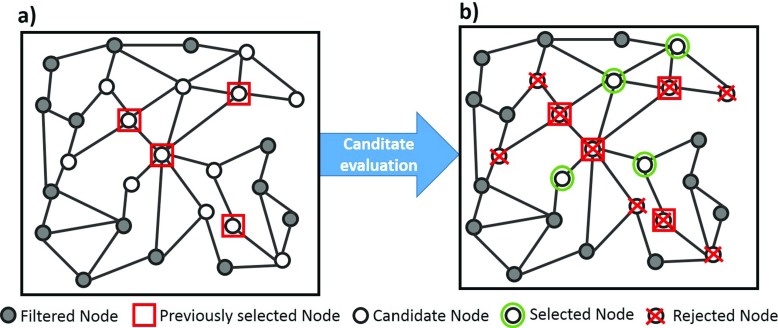
(**a**) We select the candidate Nodes (*white circles*) that connects to those used in the previous time step (*red squares*) and ignore the rest (*grey circles*). (**b**) We then evaluate these candidate Nodes using (7) to select K Nodes (*green circles*) to be used in this time step and reject the rest (*red crosses*)

The main idea is to keep track of the Filtered Nodes that have been selected to reconstruct the pose in the previous time step, and use such information to speed up the selection in the current time step. As shown in Fig. 3a, we first consider the set of candidate Nodes (white circles) that are connected to those selected in the previous time step (red squares) and ignore the rest (grey circles). This is based on the observation that human motion is continuous, and therefore the Nodes for the current time step should be relevant to those used in the previous time step. Then, as shown in Fig. 3b, we apply (7) to evaluate these candidate Nodes. The K Nodes with the best scores (green circles) are used for the current time step for pose reconstruction, and the rest of the candidate nodes are rejected (red crosses). In our system, K is set empirically as 30, as suggested in [37].

As a result, comparing with the brute force search, we only need to evaluate a small portion of the database with Equation 7 to enhance computational speed. Our algorithm also ensures that only relevant Nodes are selected, which can enhance the performance of the pose reconstruction process. Notice that for the first time step, we still need to do a full database search because the previous time step information is not available.

Computation time in [37] was *O*(*n*) where *n* is the number of poses in the database. The computation time of our system is *O*(*e*) where *e* is the average number of edge per node and *e*<<*n*, as shown in Fig. 9. Consequently, computation time is almost independent of the size of the database, which makes it possible to reconstruct poses with a large database in real-time.

### Kinect pose optimization

As described in [37] a low dimensional latent space is computed using the *K* candidate Filter Nodes which have been selected during the last step. To this end, we apply principal component analysis on the set of selected Filtered Nodes. In this space, a point is a linear combination of the selected Filtered Nodes. Optimization consists here in searching for the best combination to reconstruct the actual pose performed by the user. This is formulated as an energy minimization process with four energy terms.

The control term aims at minimizing the difference between the optimized pose *p*_*op*_ and the observed Kinect pose *p*_*k*_ while considering the reliability values of the joints. This encourages the optimized pose to be similar to the measured one at least for the reliable joints:

8 $$\begin{array}{@{}rcl@{}} Ec = mean_{j=1..j_{total}} \left[ R(j) \left( p_{op}(j)-p_{k}(j) \right) \right] \end{array} $$

The style term minimizes the difference between the optimized pose and its closest neighbour *p*_*db*_ to preserve the style of the selected pose compared to its neighbours in the database (style continuity):

9 $$\begin{array}{@{}rcl@{}} Es = \min_{db} mean_{j=1..j_{total}} \left( p_{op}(j)-p_{db}(j) \right) \end{array} $$

The bone length term minimizes the change in bone length *l*_*op*_ compared to the reference values *l*_*ref*_ to avoid unrealistic elastic body segments:

10 $$\begin{array}{@{}rcl@{}} Eb = mean_{b=1..b_{total}} \left[ (l_{op}-l_{ref})^{2} \right] \end{array} $$

Finally, the temporal continuity term minimizes high frequency jittery movements. It consists in computing the variation between the current optimized pose and the past two synthesized poses *p*_1_ and *p*_2_:

11 $$\begin{array}{@{}rcl@{}} Et = mean_{j=1..j_{total}} \left( (p_{op}(j) - 2p_{1}(j) + p_{2}(j))^{2} \right) \end{array} $$

As the pose space is highly non-linear, we apply local stochastic search to find the pose that maximizes a weighted sum of the four terms with predefined negative weights. Hence, ideal score is 0 while highly negative scores are considered to be bad:

12 $$\begin{array}{@{}rcl@{}} E = w_{c}Ec + w_{s}Es + w_{b}Eb + w_{t}Et \end{array} $$

where *w*_*c*_, *w*_*s*_, *w*_*b*_, and *w*_*t*_ are the weights. In our system, they have been set to -1.0, -0.5, -1.5 and -0.25 respectively, as suggested in [37]. The optimization process stops when a local optimal pose is found, or when the optimization takes longer than one frame time. The optimized result is back-projected to the full joint position space and form the full optimized pose.

## Physical modelling

The optimized pose obtained in Section 6 may still have some small artefacts that could affect the quality of the reconstruction, and consequently the potential user avatar animation. To tackle this problem, the optimized pose enters a dynamic filter made of a physical model of the user, as suggested in [36]. As a result, the movements of the body parts obey Newton physics, and the segments lengths are accurately maintained.

The character is represented by 19 body segments and 20 joints in accordance with the Kinect skeleton definition. The size and the mass of each segment are set according to anthropometric tables [1]. All joints are modelled as ball joints, which indicates that each segment has 3 degrees of freedom, to avoid unintentionally locking up of limbs.

We control the movement with 3 dimensional forces and the 1 dimensional torque along the body segment direction, driving the physical model to the target synthesized pose. In each time step, the control force for a joint is calculated by a PD controller with hand-tuned gains. Readers are referred to [36] for more details.

During simulation, the physical simulation engine Bullet Engine [6] maintains the segment length and segment connectivity while applying the calculated control forces and torques. The resultant pose is the equilibrium state of the character, representing the pose that can satisfy the optimized pose the most.

## Experimental results

To evaluate the performance of our system under highly constrained environments, we carried-out experiments in an industrial context under different conditions. Our proposed Filtered Pose Graph selected relevant poses before optimization, aiming at generating superior results compared to [37].

Let us consider now the experimental set-up and the results.

### Experimental setup

Ten scenarios were tested to evaluate the relevance of the real-time reconstruction method. We set-up the scenarios according to real industrial study cases where operators have to manipulate large objects during real industrial processes, leading to large occlusions. These scenarios were inspired by actual work tasks performed in car manufacturer factories. In most of these scenarios, the sensor was not placed in front of the user due to industrial cluttered environments.

**Fig. 4:**
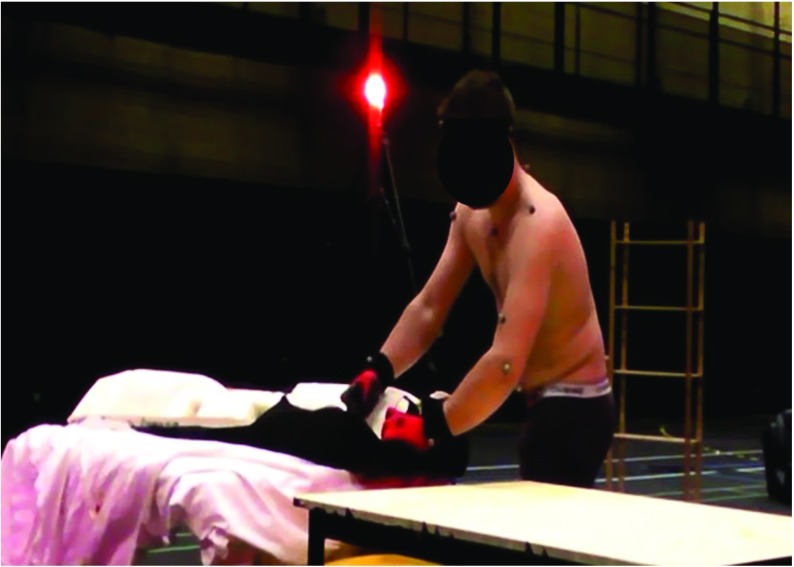
Example of in industrial environment in which the user was partly occluded by the equipment

Table 1 describes the main characteristics of the ten tested scenarios, in which we set-up three camera positions with different levels of occlusions due to external objects. Figure 4 shows an example of industrial task performed by the user when the Kinect was placed 45 ^∘^ on the left, with huge occlusions.

**Table 1:** Description of the ten scenarios used for evaluation

ID	Frames	Occlusion	Kinect Pos.	Visualization
1	1076	Few	Front	
2	1290			
3	945			
4	2385	Many	Front	
5	1488			
6	1136			
7	671			
8	1978	Many	45 ^∘^ right	
9	1968			
10	1316			

For each of these scenarios, the motion of the user was recorded by both a Kinect and a 15 cameras Vicon optical motion capture system. Synchronization between the two systems was ensured by performing a posteriori cross correlation between joint centres trajectories estimated in both systems.

In our experiments, we used a graph based on 130 motion clips of industrial activities. This resulted in 532,624 poses, which were filtered into 2,048 nodes with an average of 7.8 edges per node.

### Accuracy comparison

We compared the accuracy of poses provided by the Kinect to those reconstructed with Shum et al. [37] and our method. We evaluated the system error by comparing these poses to those captured with the Vicon optical motion capture system, supposed to be the reference system. Information about the statistical tests used in this paper can be found in [15].

Firstly, we compared the poses estimated the by Kinect (*p*_*kin*,*i*_), the pose reconstructed using [37] (*p*_*shum*,*i*_) and our method (*p*_*our*,*i*_), to the reference one (*p*_*ref*,*i*_) for each frame *i*. The error was given by:

13 $$\begin{array}{@{}rcl@{}} E_{X}\,=\,&\frac{1}{m \times n} {\sum}_{f=1}^{m} {\sum}_{j=1}^{n} | \left( p_{ref,i}(j) \,-\, parent(p_{ref,i}(j))\right) &\,-\, \left( p_{X,i}(j)-parent(p_{X, i}(j))\right) | \end{array} $$

where *X* stands for the method, *m* is the number of frames, *n* is the number of joints and *p* *a* *r* *e* *n* *t*(*p*_*i*_(*j*,*f*)) returns the parent node of *p*_*i*_(*j*,*f*) in the human skeleton hierarchy.

**Fig. 5:**
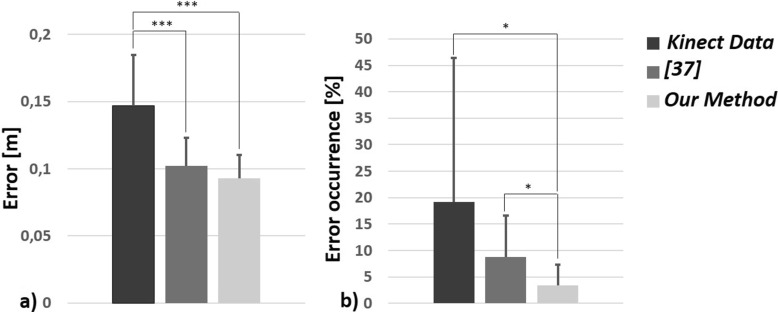
Accuracy analysis for Kinect (*dark grey*), [37] (*medium grey*), and our method (*light grey*) with (**a**) mean errors, and (**b**) percentage of frame with error greater than 0.2m

We evaluated the mean error for each method and its standard deviation (*σ*) across all scenarios. Kolmogorov-Smirnov test was used to check the normality of the distribution. We found that the mean error followed a normal law. An Anova test was used for a one-way repeated measures analysis of variance. A Bonferroni post-hoc test was conducted to detect significant differences between methods. The level of significance was set to *p*<0.001, denoted with *** in Fig. 5a. The mean errors of [37] (0.10m, *σ*=0.02) and our method (0.09m, *σ*=0.02) were significantly (*p*<0.001) lower than those of the Kinect (0.15m, *σ*=0.04). Our method performed slightly better than [37].

Secondly, we calculated the percentage of frames in which the error was greater than or equal to 0.2m, *E* *r* *r*0.2_*X*_. Such a large error would create noticeable visual artefact that would impact the user experience in immersive/interactive applications. Incoherence between the user pose and the immersive feedback may heavily distract the attention of the user when performing a task in such interactive applications.

We evaluated the means, standard deviations (*σ*), min and max of *E* *r* *r*0.2_*X*_ across all scenarios. The distributions did not follow a normal law. A Friedman test was used for a one-way repeated measures analysis of variance. A Wilcoxon signed rank post-hoc test was carried-out to detect significant differences between methods. The level of significance after Bonferroni correction was set at *p*<0.01, denoted with * in Fig. 5b. There were significantly (*p*<0.01) less cases where the error was greater than or equal to 0.2m between Kinect *E* *r* *r*0.2_*kin*_ (19.1 %, *σ*=27.3, *m* *i* *n*=0.3, *m* *a* *x*=92.1) and our method *E* *r* *r*0.2_*our*_ (3.3 %, *σ*=3.9; *m* *i* *n*=0, *m* *a* *x*=10.5). However, while the value of [37] *E* *r* *r*0.2_*shum*_ (8.8 %, *σ*=7.9, *m* *i* *n*=0, *m* *a* *x*=19.7) was lower than those of the Kinect *E* *r* *r*0.2_*kin*_, no statistical difference has been shown. There was significant (*p*<0.01) improvement between *E* *r* *r*0.2_*our*_ and *E* *r* *r*0.2_*shum*_ supporting the hypothesis that the Filtered Pose Graph used to preselect pose candidates actually enhance the performance of the reconstruction method.

Thirdly, to further study the performance of each method, we studied the histogram of errors represented as the percentage of frames in quantized error bands. In most immersive/interactive applications, the occurrence of large errors is more problematic than small ones, as the user would experience noticeable artefacts.

**Fig. 6:**
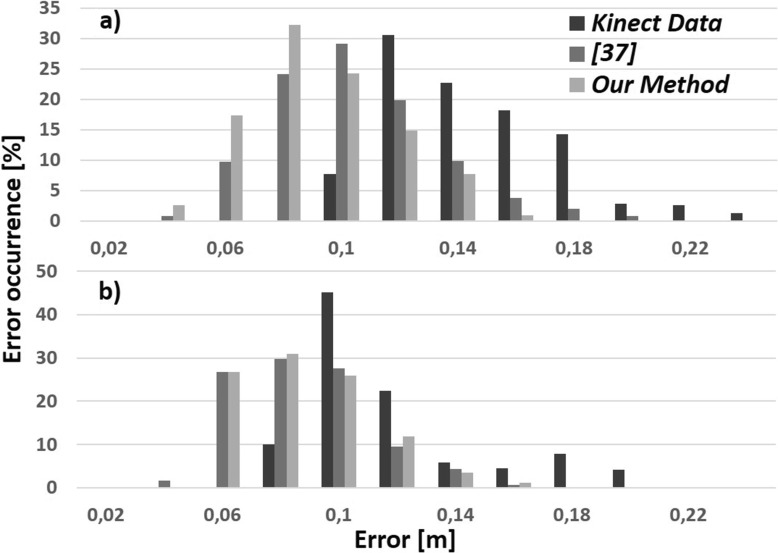
Percentage of error occurrence (in %) for each level of error (in m), between the Kinect and Vicon data, for Kinect (*dark grey*), [37] (*medium grey*), and our method (*light grey*) in (**a**) scenario 8 and (**b**) scenario 10

In Fig. 6, we analysed two of the most challenging scenarios more precisely: 8 and 10 in which the operator manipulated car seats leading to large occlusions. Scenario 8 was about fitting the protective casing of the seat. Scenario 10 was about in screwing the seat adjustment handle. As a result, in scenario 8, the mean error of our system *E*_*our*_ (0.08m, *σ*=0.02) and previous work [37] *E*_*shum*_ (0.09m, *σ*=0.03) were similar. However, as shown in Fig. 6a, our system had a much better error distribution, which is shifted towards the lower error value levels, compared to those using previous work [37]. Our system performed the worst in scenario 10, in which the mean error *E*_*our*_ (0.078m, *σ*=0.02) was slightly higher than those of [37] *E*_*shum*_ (0.076m, *σ*=0.02). As shown in Fig. 6b, our method had similar error distribution as previous work [37], and shifted the Kinect error from high value bands to lower ones. Except scenario 10, our method had better error distribution compared to previous work [37] in all other scenarios.

**Fig. 7:**
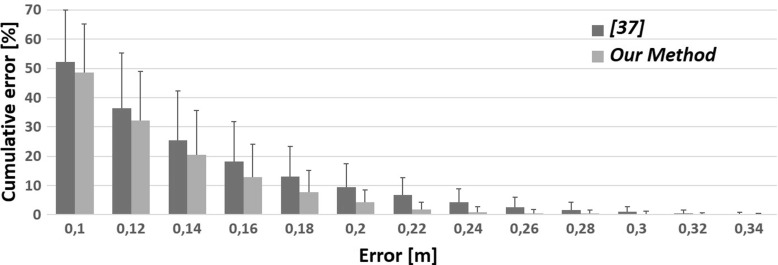
Histogram of cumulative errors in our method (*light grey*) and [37] (*medium grey*)

Fourthly, to quantify the ability to avoid large errors for each method, we computed the number of cases for which the error was greater than a set of error values in all scenarios,. The results are shown in Fig. 7. Our method consistently achieved lower error values in all bands. More importantly, in larger error bands, the difference between our method and previous [37] became larger. This was mainly due to using the Filtered Pose Graph during the pose selection phase as most of the other parts of the methods were similar.

**Fig. 8:**
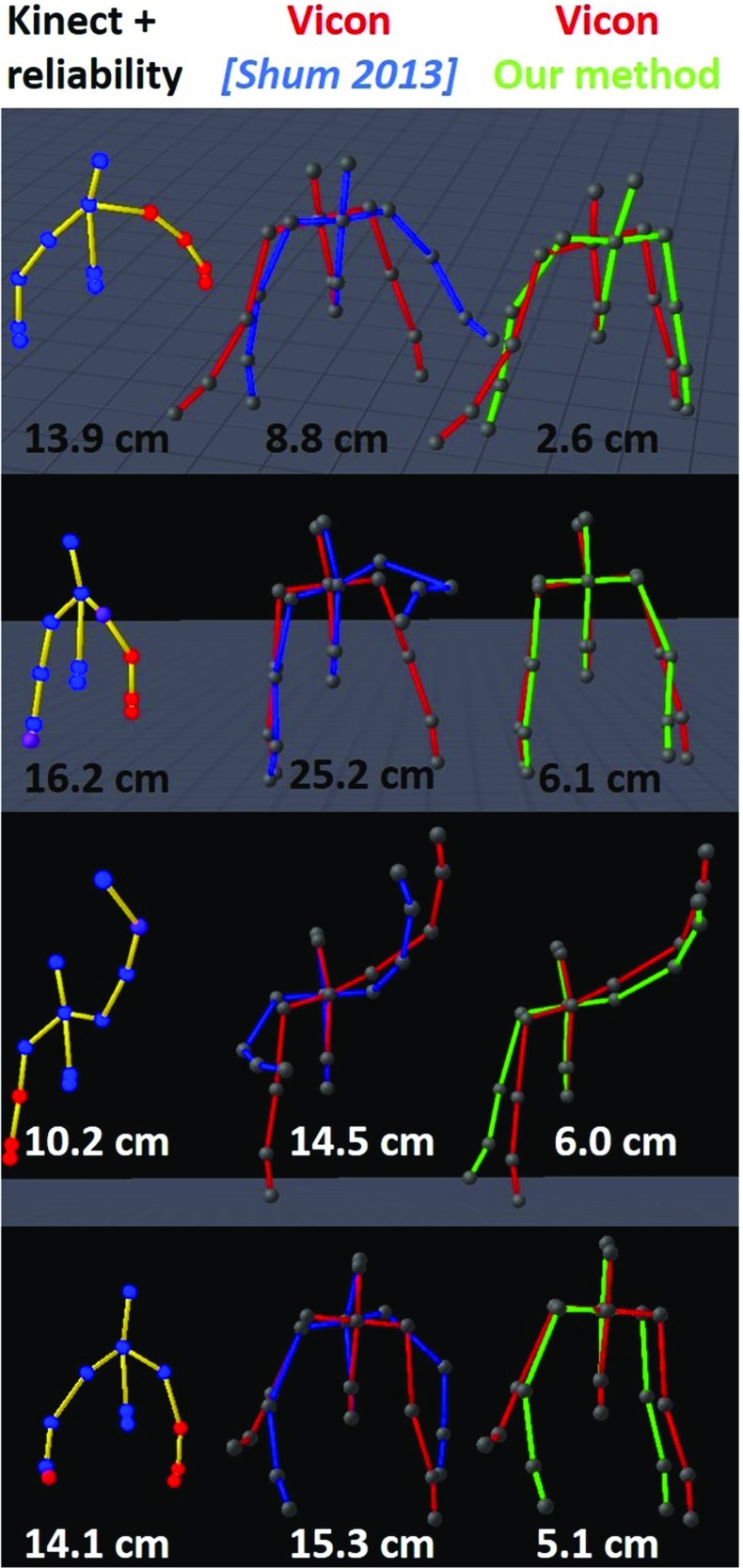
Comparison of reconstruction performance on challenging poses

Finally, we illustrated several challenging poses in which high errors occurred, as shown in Fig. 8. For each of the four poses, we showed the Kinect pose on the left, the reconstructed pose using previous work [37] in blue, the reconstructed pose by our method in green, and the reference one in red. We calculated the error between each pose and the reference one for each frame using (13) (in cm), as depicted in Fig. 8 below the poses. Our method always overperformed previous work [37]. As our method is able to produce continuous poses even if only few reliable information is given by the Kinect, it is well adapted to situations with large occlusions. Hence, using an unorganized database of examples could lead to discontinuous poses that rapidly diverge from the actual trajectory, while continuity ensures local correspondence with the actual trajectory.

A supplementary video is attached to this paper to assess the quality of the reconstruction in these scenarios.

### Performance analysis

In these evaluations, our system ran faster than real-time (i.e. 30Hz). The average frame time of our method was 12.28ms (*σ*=0.7), which is lower than those based on using previous work [37] 14.53ms (*σ*=1.0). This is mainly due to the preselection of successors in the Filtered Pose Graph that limits the number of candidates and provides more relevant examples. The offline computation time to design the Filtered Pose Graph used in these experimentations was 115*s*.

**Fig. 9:**
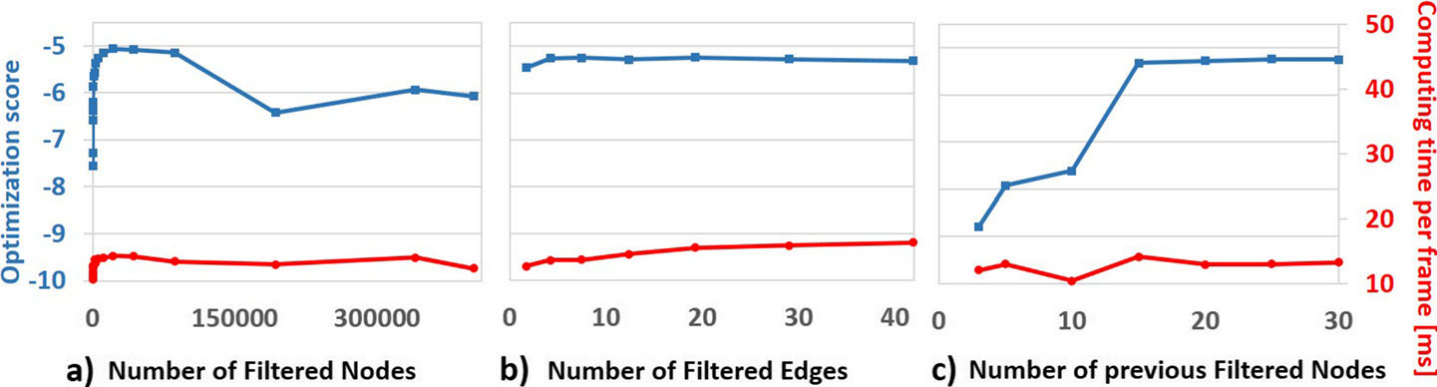
The optimization score (*left axis, blue line*) and computing time per frame in ms (*right axis, red line*) for different number of (**a**) Filtered Nodes in the Filtered Pose Graph, (**b**) Filtered Edges per Filtered Node, and (**c**) Filtered Nodes selected for reconstruction

We analysed the effect of the Filtered Pose Graph parameters on reconstruction quality and computational time. The results is shown in Fig. 9. The optimization score is calculated according to (12).

As shown in Fig. 9a, a heavily filtered pose graph using high threshold *t* *h* *r* *e* *s*_1_ led to poor reconstruction quality, due to the small number of relevant poses available for preselection. We found better results when using at least 2,000 nodes in the Filtered Pose Graph. In contrast to [37], the number of nodes in the graph did not directly affect computation time. This is due to the efficient pose selection process based on the Filtered Pose Graph.

As shown in Fig. 9b, the number of Filtered Edges per Filtered Node had minimum effect on the performance and computation time. There was only a small increase in computation time due to the evaluation of a slightly larger number of connected nodes.

As shown in Fig. 9c, the number of nodes used for reconstruction (*K* value in Section 6.2) strongly affected the optimization score. Using 15 Filtered Nodes, the reconstruction quality reached a plateau and computation time per frame remained constant.

Overall, the results showed that the Filtered Pose Graph allowed us to use large database of examples while maintaining good performance. Thus it could be possible to extend the database with more examples, to address a wider variety of motions, without strong impact on computation cost.

## Conclusion

The main contribution of this paper is to introduce a new data structure to enhance the performance of algorithms aiming at reconstructing unreliable Kinect skeleton data in cluttered environments. The Filtered Pose Graph structure helps to preselect a set of relevant poses in a very efficient manner. Our results showed a lower average error compared to those obtained with previous works that used an unorganized database. In many cases, especially with large errors, our method achieved better performance thanks to the more efficient pose selection process.

If extreme occlusion persists during a long time, the reconstructed poses may diverge from the actual trajectory because too few reliable information is available. Our system tackle the problem by including criteria focusing on unreliable joints to select a set of relevant poses for reconstruction. This allows us to outperform previous works [37]. In some extreme situations, it would be interesting to consider using multiple Kinects, but it would require to calibrate all the sensors, which is not always possible in wide public applications.

One of our future direction is to improve the reliability terms to avoid false positive and negative values. In an industrial context, tasks are usually repetitive and performed with a logical sequence. We will explore reliability terms that exploit the prior knowledge of industrial tasks. We are also interested in adapting the framework to other serious applications such as sport analysis and rehabilitation using immersive environments, especially when real-time feedback with avatars is needed. As our system is more robust to occlusions, we should be able to achieve better presence in virtual reality applications, but further perception studies are needed to evaluate the actual impact.
